# Moving, Seeing, Hearing, Smelling and Tasting: How Sensory–Motor Experiences Shape Early Cognitive Development

**DOI:** 10.3390/bs16020255

**Published:** 2026-02-10

**Authors:** Chi-hsin Chen, Claire D. Monroy

**Affiliations:** 1 Department of Psychology, University of Liverpool, Liverpool L69 7ZA, UK; 2 School of Psychology, Keele University, Keele ST5 5BG, UK

**Keywords:** sensory experiences, early cognitive development, motor experiences, vision, hearing, smell, taste

## Abstract

In the past few decades, we have seen increasing specialization within developmental science, with researchers focusing on narrowly defined research areas in child development. This specialization has yielded deep insights and methodological advances across many developmental areas. However, it has also led to siloes of expertise. In this article, we review findings on how motor, visual, auditory, olfactory and gustatory experiences affect early cognitive development. We identify some common themes across these domains, such as the role of predictive processing in early development. We argue for the importance of adopting a dynamic systems approach and considering the variabilities both within the individual and in the larger cultural environments. Finally, we conclude by outlining several avenues for future research that seek to advance integrative approaches within developmental science.

## 1. Introduction

The study of human cognitive development has undergone a significant evolution from an early emphasis on the individual child (e.g., Piaget, 1962) to a broader consideration of the child embedded within social and environmental contexts (e.g., Bronfenbrenner, 1979). Contemporary theoretical frameworks such as the dynamic systems (Thelen & Smith, 2007) and developmental cascade theories (Masten & Cicchetti, 2010; Schneider & West, 2025) underscore the interdependent and coordinated nature of skill acquisition across sensory domains. These perspectives assert that emergent abilities in infancy cannot be fully understood in isolation but must be examined as part of a dynamic, interacting system.

The term “whole child” approach is frequently invoked to capture this holistic perspective, emphasizing the necessity for researchers to adopt a broad, integrative view of development. However, the increasing specialization within developmental science—both conceptually and methodologically—has led to siloes of expertise. While such specialization is essential for cultivating deep knowledge within specific domains, it can inadvertently hinder progress toward a comprehensive understanding of the developing child.

In this review, we explore the role of sensory and motor experiences in shaping cognitive development. For the purposes of our review, we use the term “sensorimotor” to refer to the integration of sensory input with motor output, while “sensory–motor” refers to sensory and motor experiences collectively. Our aim is to bring together traditionally siloed areas of research by examining how experiences in each sensory–motor modality contribute to cognitive growth. This review is not aimed at comprehensively and objectively cataloguing all available evidence across all sensory modalities in child cognitive development, which would lie far beyond the scope of a single paper. Rather, our aim is to offer a broad perspective that may help to highlight patterns across modalities, which could inform and enrich our individual lines of inquiry.

We therefore conducted this review using a targeted search strategy. We prioritized empirical papers that presented evidence for links between one sensory or motor modality and a cognitive domain. We also consulted prior narrative and systematic review papers on relevant topics. Our search strategy focused on studies asking research questions based on each modality that we preselected. For example, does motor development affect cognitive development? Does vision or lack of vision affect cognitive development? We conducted our search by using search keywords such as “motor development and cognitive development”, “vision and cognitive development”, “blindness and cognitive development”. Google scholar was the primary database used for searching for articles. We used the “sort by relevance” feature to scan the first 200 to 300 relevant search results for each round of keyword search. Relevant papers were scanned by reading the abstract, and if the topic met our criteria, then we read the paper in full. Therefore, our search strategy involves the objective search criteria implemented by Google Scholar’s “sort by relevance” feature and subjective judgement made by us on whether a paper was relevant to our topics. Additional papers were identified by searching within the reference sections of included papers, particularly topical review papers, as well as papers that cited them. We included empirical articles, review papers, and book chapters in our review. Papers were excluded if they did not focus on the relationship between a specified sensory domain and a cognitive process(es) (e.g., “The development of vision”, “Color vision in children and the Lanthony New Color Test”). We also excluded studies that focus on intersensory, multisensory, or cross-modal sensory processes, which were outside the scope of this review. While we aimed to include the most recent evidence available, we did not restrict our search to a certain time window.

After finding papers relevant to our review topics, we then read through the papers and identified a few lines of research to focus on for each sensory–motor modality. Our goal was to present a selective review that highlighted the relationship between sensory–motor experiences and cognitive development, rather than providing a systematic and comprehensive review. We therefore do not focus on how different designs, measures, sampling methods, and effect sizes affect the outcomes or interpretations of different findings. Instead, we focus our effort on finding common themes across domains and identifying understudied areas that warrant further research. For the readers that are interested in systematic reviews or critical reviews, we have listed a few in Table 1 for reference.

In this review, we begin with the motor domain, examining how early motor experiences contribute to the foundations of cognitive development. We will then consider vision, audition, olfaction, and finally gustation. We will conclude with a discussion of some overarching themes and remaining questions for future research to address.

## 2. Motor Experiences

The idea that motor experiences play an important role in cognitive development is not new. According to Piaget (1962), during the first two years of life, a period he called the sensorimotor stage, infants rely on their sensory input and motor actions to interact with and understand the world. They learn to coordinate their sensory experiences and motor movements. Through that coordination, they build concepts about the world.

In this section, we will focus on how motor experiences influence cognitive development. The discussion will be divided into three parts. We will start by devoting the focus of the section on how motor development in infancy (and toddlerhood) may affect cognitive development—either directly by influencing how infants interact with the world, or indirectly by impacting how social partners interact with infants. In the second section, we will discuss how motor learning during preschool and school years may facilitate cognitive development. Following the distinctions made by Pesce et al. (2019), we refer to motor development when we discuss the developmental changes in motor behaviors that are potentially related to survival, such as sitting or walking. On the other hand, we refer to motor learning when we discuss motor behavioral changes that are decoupled from survival, such as catching or hitting a ball or dancing. In the last part, we will discuss how cultural practices may influence motor development and the implications for cognitive development.

### 2.1. Motor Development Creates Opportunities for Cognitive Development

Motor development appears to influence cognitive development in two ways—directly, by changing the information created by the infants themselves, or indirectly, by changing the information provided by social partners to infants. First, better motor skills (such as sitting, crawling, or walking) allow infants to interact with the world differently and sample, store, and use information in different ways. In one study with 50 infants observed at the ages of 11 and 13 months, Karasik and colleagues (Karasik et al., 2011) found that, compared to crawling infants, walking infants were more likely to access distant objects, carry objects, and approach mothers to share objects. With better motor skills, infants develop different and (and potentially more informative) exploratory behaviors, have more flexible memory, and are better at using landmarks in their navigation (Clearfield, 2004; Herbert et al., 2007; Soska & Adolph, 2014). Second, infants’ motor development also changes how caregivers or social partners interact with them and thus changes the information they receive during social interactions (Karasik et al., 2011, 2014; Kretch et al., 2022, 2023).

Infants tend to engage in different object exploration behaviors, such as fingering, tapping, looking, and mouthing, when they are sitting, which requires more advanced posture control compared to supine or prone (Soska & Adolph, 2014). Importantly, infants engage in more sophisticated, multi-modal object exploration (e.g., simultaneous looking and manual exploration) when they are sitting than when they are supine or prone. Multimodal object explorations may allow infants to better recognize objects from different angles, which is a critical step for object concept building (Bambach et al., 2016; James et al., 2014). Multimodal object explorations also sustain infants’ attention during object play and facilitate the learning of object names and object concepts (Chen et al., 2021; Schroer & Yu, 2023; Suarez-Rivera et al., 2019; Yu & Smith, 2012).

In addition to object exploration, which leads to better object recognition and learning, children with more advanced motor development also show more flexible memory (Herbert et al., 2007), better mental rotation ability (Schwarzer et al., 2013), and better use of landmarks in their navigation (Clearfield, 2004). For example, Herbert et al. (2007) tested a group of 9-month-old infants (*n* = 96) and found that crawling infants had more flexible retrieval and better generalization of action events than pre-crawling infants. This difference is likely driven by the fact that independent locomotion provides infants with more opportunities to retrieve memories in different contexts. Neurophysiological evidence also shows that infants’ motor system is more strongly activated when they observe an action with which they have more experience (e.g., crawling vs. walking), which suggests that infants’ own motor experiences change their action perception (van Elk et al., 2008). Furthermore, Schwarzer et al. (2013) showed that compared to pre-crawlers (*n* = 24), crawlers (*n* = 24) were better able to distinguish a familiar object from its mirror image, which indicates superior mental rotation and object recognition abilities. As the above-mentioned studies did not directly test the causal effects of infants’ locomotion on their memory and mental rotation abilities, the relationships found in those studies may best be viewed as correlational. However, a recent study (Schwarzer et al., 2022) recruited 30 pre-locomotor 6-month-old infants and randomly assigned them into either a locomotion training group or a control group. The infants in the training group received self-produced locomotion by using a wheeled-walker. After training, these infants were significantly better at distinguishing an object from its mirror image when compared to their own pre-training performance and to the control group. This study established the direct links between infants’ locomotion and mental rotation and object recognition abilities.

Motor development also influences infants’ spatial cognition by changing how they use landmarks in their navigation. Clearfield (2004) examined the relationship between infants’ (*n* = 72 in two experiments) expertise in crawling and walking and their use of landmarks to find a hidden goal (i.e., their mothers) in a large space. Compared to expert crawlers and expert walkers (i.e., infants with several months of crawling or walking experiences), novice crawlers and novice walkers were less able to use landmarks to find their goal locations. Interestingly, novice walkers, who were also usually expert crawlers by experience, did not seem to completely transfer their knowledge or landmark using ability from their prior crawling experience into their relatively new walking experience. However, walkers seemed to require less time to master the use of landmarks compared to crawlers. Crawling infants needed at least 12 weeks of crawling experience to consistently solve spatial search and landmark use problems. In contrast, walking infants only needed 7 weeks of walking experience to reach perfect performance. This indicates that novice walkers may not start from scratch and do not need to relearn everything about spatial search and landmark use when they start walking. This finding is in line with studies on infants’ recognition of surface affordances. Adolph and colleagues (Adolph, 1997; Kretch & Adolph, 2013) found that novice walkers did not seem to transfer their knowledge of surface properties from their crawling experience.

Findings from these different studies suggest that cognitive development is embodied and embedded in the environment (Adolph & Hoch, 2019; Adolph & Robinson, 2015; Musculus et al., 2021). These findings support the view that children’s understanding of the world and their ability to use different knowledge and cognitive skills, are not abstract but instead are greatly affected by their body experiences *in the environment*. Additional evidence of embodied cognition can be found in older children. For example, 4- to 12-year-old children’s (*n* = 90) ability to physically move from one location to another, as opposed to imagining the move, facilitates their performance in perspective taking tasks where they need predict the visual information they will receive from a different visual perspective (Hirai et al., 2020). Moreover, bodily changes affect children’s motor planning abilities, which in turn impact their cognitive planning abilities (i.e., the ability to think about action sequences to efficiently plan a task, see the review in Musculus et al., 2021).

Infants’ motor development also affects the interactions they have with social partners. For example, infants who can sit independently spend more time facing their caregivers and handling objects than infants who need support to sit (Kretch et al., 2023). Caregivers provide more cognitive learning opportunities (e.g., encouraging infants to explore objects visually or manually, or engaging in joint object play) when infants sit independently compared to when they are supine, prone, or need supported sitting (Kretch et al., 2022). Similarly, a recent study (Rousey et al., 2026) using daylong home recording with 64 infants (aged 4–7 months and 11–14 months) found that increased time in supine and prone positions predicted fewer adult words that infants hear while increased time in sitting and being held predicted increases in adult word count. Another recent study with typically developing infants (*n* = 28) and infants with cerebral palsy (*n* = 22) found that caregivers provide more object labels to infants when they are sitting compared to other postures (e.g., supine or prone, Kretch et al., 2025). However, infants with cerebral palsy received fewer object labels compared to their typically developing peers. In addition, walking infants are more likely to make moving bids (i.e., moving and showing or offering an object to a caregiver) while crawling infants often must stop crawling to make stationary bids (i.e., showing or offering an object to a caregiver while remaining in a place (Karasik et al., 2011, 2014). Moving bids and stationary bids elicit different verbal responses from caregivers (Karasik et al., 2014). These studies provide converging evidence that infants’ motor development changes the information they receive from their social partners.

Interestingly, autonomous locomotion seems to affect infants’ expression of anger when their goals are blocked (Campos et al., 1992; Roben et al., 2012). In a longitudinal study conducted by Roben et al. (2012), mothers reported that, after they started crawling, their infants (*n* = 20) showed more angry emotions when being restrained, compared to before they had begun crawling. According to the authors, emotions “serve a purpose in helping individuals achieve their goals in various contexts” (p. 560) and “anger is an approach-oriented emotion that supports action in response to a blocked goal” (p. 565). Altogether, these findings suggest that motor development affects infants’ social and emotional responses, and how their social partners interact with them.

### 2.2. Motor Learning Provides Training Ground for Cognitive Development

Beyond the first few years of life, motor experiences gained from motor learning during preschool and school years may also influence cognitive development. Studies using different motor skill measures (e.g., bilateral body coordination vs. fine motor skills) and cognitive skill measures (e.g., short-term memory vs. fluid intelligence) have led to different conclusions (for a detailed discussion, see van der Fels et al., 2015; Zeng et al., 2017). However, research with both typically developing children aged between 4 and 18 and children with motor difficulties has shown positive correlations between complex motor skills (e.g., fine manual control, coordination of movements in rhythm, or movements in sequence) and higher order cognitive skills, such as visual processing or fluid intelligence, which are involved in problem solving (see correlational studies in Davis et al., 2010, 2011; Jenni et al., 2013; as well as systematic reviews such as Best, 2010; van der Fels et al., 2015; Zeng et al., 2017). Studies with some clinical populations, including children with autism spectrum disorder (ASD), attention deficit hyperactivity disorder (ADHD), dyslexia, and developmental coordination disorder (DCD) have shown concomitant delays in motor development and cognitive development (Davis et al., 2011; Diamond, 2000; LeBarton & Iverson, 2016; Shimko & James, 2025; Wilson et al., 2013). It has been suggested that one reason why we see close relations between motor and cognitive assessments is related to the fact that cognitive tasks which require the prefrontal cortex often also activate the cerebellum, which is also involved in the acquisition of motor skills (Diamond, 2000; Koziol et al., 2014).

Several researchers have argued that motor learning, particularly during aerobic exercise, may contribute to cognitive functions, such as executive functions, via three pathways: (1) by providing the experiences of engaging in cognitively demanding, goal-directed activities (e.g., group exercises that require cooperating with teammates or anticipating teammates’ or opponents’ behaviors), (2) by providing the experience of learning and executing complex movements (e.g., dancing), or (3) by inducing physiological changes, such as increasing cerebral blood volume or neurochemicals (Best, 2010; Pesce et al., 2019). One implication of these findings is that motor learning could be considered in treatment or intervention programs to facilitate cognitive development, although empirical validation is needed (Best, 2010; Davis et al., 2010, 2011; Pesce et al., 2019; van der Fels et al., 2015).

### 2.3. Cultural Differences Affect Motor Development

Despite the wealth of research on how motor experiences may affect cognitive development, most of the studies were conducted in the Western, Educated, Industrialized, Rich, and Democratic (WEIRD) cultures (Adolph & Hoch, 2019; Adolph & Robinson, 2015). It is not clear whether the same findings may apply to other cultures. Different cultures have different expectations, practices, or routines that may affect motor development (Adolph & Hoch, 2019; Adolph & Robinson, 2015; Hopkins & Westra, 1990; Karasik et al., 2015, 2023). For example, for infants raised in a Jamaican community where independent sitting and walking are valued (as opposed to crawling) and facilitated by caregivers’ use of massaging and passive stretching movements, they tend to show earlier sitting and walking abilities compared to infants not living in that community (*n* = 29 in the former and group and *n* = 26 in the latter group; Hopkins & Westra, 1990). Five-month-old infants living in cultures where it is viewed as safe or appropriate for infants to sit on adult furniture (e.g., Kenya or Cameroon) spend more time sitting independently on the furniture with their mothers out of reach, compared to 5-month-olds living in the US, who often sit on infant chairs or need supported sitting (72 infants in total, with 12 from each of the six countries studied; Karasik et al., 2015).

Karasik et al. (2023) found that, in Tajikistan, where gahvora cradling practices severely restrict infant movements for extended time periods, infants (*n* = 269) showed delayed sitting, crawling, and walking compared to the World Health Organization (WHO) standards. But by the time Tajik children are 4 to 5 years (*n* = 91), their motor skills are comparable to children in the US. The authors suggested the interim opportunities to practice different motor skills in early childhood may have allowed Tajik children to catch up, as these children often engage in activities and display motor skills that are not usually included in standard assessments, such as navigating different terrains, carrying heavy loads, or climbing tall ladders. These findings reflect developmental plasticity—structures, abilities, and skills developing or adapting in response to different experiences (Oakes, 2017).

These studies illustrate that cultural practices may affect early motor development. Motor development in infancy and toddlerhood may influence children’s learning opportunities by directly affecting how children interact with the world and indirectly affecting how caregivers interact with children. However, it is still an open question whether the differences in early motor development have cascading effects on early cognitive development, beyond milestone attainment and the trajectories in how children achieve those milestones. Motor learning or training in the preschool and school age years can potentially facilitate or improve cognitive functioning and may open up a way to study developmental plasticity. Does developmental plasticity simply reflect the product of different developmental pathways experienced by children living in different cultures or different learning environments? Can these products create new inputs and shape further processes and structures in subsequent development (Oakes, 2017)? These questions are worthy of further investigation.

## 3. Vision

Compared to other senses, vision provides high bandwidth and precision of information intake (Cappagli et al., 2017; Giudice, 2018; Pasqualotto & Proulx, 2012; Röder et al., 2004; Tinti et al., 2006). For example, it allows simultaneous intake of huge amounts of information (e.g., seeing many things at the same time), which contrasts with the sequential information intake through touch (e.g., touching one thing or a small area at a time; Giudice, 2018; Pasqualotto & Proulx, 2012; Tinti et al., 2006). Vision tends to provide better precision of object locations compared to hearing, which often only allows us to get the general direction of a sound source (Giudice, 2018). Vision also offers a frame of reference for multisensory spatial representation (Cappagli et al., 2017; Röder et al., 2004).

It has long been found that early visual deprivation, even for a few days or a few months, affects both low- and high-level perceptual processing (see Maurer et al., 2005, for a review). For example, pre-term infants who underwent 7-day phototherapy for hyperbilirubinemia (*n* = 14), and therefore had their eyes covered the whole time during that period, showed reduced preference for face-like stimuli (de Almeida et al., 2025). This decrease in preference is significant when compared to their own baseline (i.e., prior to phototherapy) and compared to a control group preterm infants that did not receive phototherapy (*n* = 15). In addition, individuals born with bilateral or unilateral cataracts that were later removed between two and six months of age showed atypical face perception (Geldart et al., 2002; Le Grand et al., 2001, 2004). They are less able to discriminate between individual faces differing in the spacing of internal facial features (Le Grand et al., 2001, 2004). They also have more difficulties recognizing a face from a different point of view (Geldart et al., 2002). These studies suggest the critical timing of visual input in the first few months of life and point to the sensitive periods in visual development.

While these are small clinical studies with limited sample sizes, there is converging evidence that, in addition to perceptual processing, early visual deprivation affects several other areas of early development. In the following, we will first review studies showing that early blindness can affect an area that, at the first glance, may seem to have little relevance to the use of vision—children’s motor development. Differences in motor development can then play a role in shaping blind children’s cognitive learning opportunities; this thus creates an indirect influence of vision on cognitive development. Following that, we will then discuss how blindness may affect spatial cognition and social interactions. In the last section, we will discuss a few areas where blind individuals show equivalent or even better performance than sighted individuals.

### 3.1. Lack of Visual Experiences Affects Motor Development

Lack of vision does not only affect motor development in aspects that require visual information, such as grasping an object, but also in aspects where vision does not seem to be directly relevant, such as posture control or initiating a change in posture (Prechtl et al., 2001; Tröster et al., 1994; Tröster & Brambring, 1993). Many fine-motor tasks, such as grabbing a moving object or grasping a small item, require infants or young children to align what they see with their own hand movements. For sighted children, they can use visual feedback to constantly update the trajectory of their own movement. However, for blind children to grasp a moving object, they often need to rely on the sound—which tends to be transient and much less reliable than visual information—to localize the object.

Relying on sound, particularly transient sound, to localize an object requires higher memory demands and better prediction of object movement (Bigelow, 2003; Tröster et al., 1994; Tröster & Brambring, 1993). The higher memory and computational demands involved in blind infants’ tracking and grasping of sounding objects may be one reason blind children often show delays in their fine motor development (Tröster et al., 1994; Tröster & Brambring, 1993). On a relevant note, reaching for sounding objects has been proposed to require the understanding that an object continues to exist even out of sight; and thus, this behavior has been used as a critical indicator of blind infants’ object permanence concept (Bigelow, 2003; Fazzi et al., 2002, 2011). Due to the higher memory and computational demand to keep objects in mind, blind infants show delays for several months to over a year in their development of object permanence concepts assessed using different tasks, relative to sighted infants (Fazzi et al., 2011; Rogers & Puchalski, 1988).

In addition to fine motor skills that require visual input, blind infants also show delays in motor skills in which visual information does not seem critical at first sight, such as posture control or initiating posture changes (Prechtl et al., 2001; Tröster et al., 1994; Tröster & Brambring, 1993). According to Tröster and Brambring (1993), the lack of vision leads to delays in the development of those motor skills because visual information provides objective cues (e.g., vertical vs. horizontal information), helps fine-tune vestibular-proprioceptive activities, and allows for the monitor and stabilization of body movements and positions (see also Peterka, 2018).

Another important reason for blind infants’ delays in posture control and change may be motivational (Tröster & Brambring, 1993). For sighted infants, movements such as raising the head while supine, turning the head, changing body postures all allow infants to see from a different perspective and provide different visual information (Franchak et al., 2011; Kretch et al., 2014), which is in itself rewarding (Gottlieb et al., 2013; Kidd & Hayden, 2015; Oudeyer & Smith, 2016). In contrast, for blind infants, changing postures or body positions does not provide much gain in terms of the external information they receive. This may be one of the reasons why, compared to sighted infants, blind infants have been found to remain in the same posture for a longer period and are less likely to initiate a change in posture in some observational studies (Tröster & Brambring, 1993). Another reason for not initiating posture changes is to avoid bumping into obstacles, which may also explain why some blind infants skip crawling before walking or crawl backwards for the first few weeks of crawling (Tröster et al., 1994). These are likely ways to avoid knocking the head against obstacles in the crawling movement, as it is harder to use the hands to identify and avoid obstacles when crawling.

As can be seen in the section on Motor Experiences of the present article, different motor experiences provide infants with different learning opportunities and different ways to interact with the world (Kretch et al., 2014, 2022, 2025; Roben et al., 2012; Soska & Adolph, 2014). One important thing to consider is how these blind infants’ different motor experiences may affect how they explore their environment and create different learning opportunities for the young developing minds, which could have cascading effects on their cognitive development.

### 3.2. Lack of Visual Experiences Affects Social Interaction and Social Cognitive Development

Lack of vision may also influence social development in several ways. For sighted infants, they pay attention to their social partner’s facial expressions (e.g., a smile) and use that information to guide behaviors, such as deciding whether to approach a stranger or to cross a seemingly dangerous surface (Striano & Rochat, 2000; Vaish & Striano, 2004). Even though blind infants have access to the tone of voice of a social partner, they receive far less information about their partner’s emotional state compared to sighted infants (Gamond et al., 2017; Grumi et al., 2021; Klinge et al., 2010). In addition to the differences on the receiving end, many studies suggest that blind infants show fewer contact-seeking behaviors or facial expressions and are less likely to initiate interactions, compared to their sighted peers (Campbell & Johnston, 2009; Freeman et al., 1989; Grumi et al., 2021; Gui et al., 2023; Tröster & Brambring, 1993). When being touched or spoken to, they sometimes freeze and stop moving and vocalizing (Tröster & Brambring, 1993). Parents often need to be more explicit about their feelings and intentions and tend to be more directive when interacting with blind infants (Campbell & Johnston, 2009; Grumi et al., 2021).

In addition to social interaction differences with caregivers, compared to sighted peers, congenitally blind children have shown a delay in their development of joint attention skills in early development (Bigelow, 2003). As they get older, they also tend to show delays in passing theory of mind tasks, such as visual-perspective taking tasks or misleading appearance and changed locations tasks (Begeer et al., 2014; Peterson et al., 2000; Richardson et al., 2023; Roch-Levecq, 2006). For example, while sighted children often pass the misleading appearances or changed locations tasks by 4 or 5 years of age, blind children often do not completely master these tasks until about 11 or 12 years of age (*n* = 23; Peterson et al., 2000). Interestingly, when performing theory of mind tasks, blind children show activations in similar brain regions, but these are quantitatively weaker compared to their sighted peers (17 blind and 114 sighted children; Richardson et al., 2023). It has been argued that, to compensate for the lack of visual input, blind children need to rely more on language to develop social pragmatic skills than their sighted peers (Bigelow, 2003).

### 3.3. Visual Experiences Affect How Individuals Code and Represent Space

Early visual deprivation greatly affects spatial cognition, particularly how individuals code the spatial relations between the self and the surrounding environments or between objects (Battal et al., 2020; Bleau et al., 2023; Giudice, 2018; Ottink et al., 2022; Pasqualotto & Proulx, 2012; Tinti et al., 2006; Ungar, 2018). Even though we receive spatial information from different senses, such as vision, hearing, and touch, vision generally provides more precise and a larger amount of spatial information than other senses (Cappagli et al., 2017; Giudice, 2018; Ottink et al., 2022). For example, vision often provides more precise information about self-to-object distances, inter-object relations, and the global structure of the environment (Giudice, 2018; Ottink et al., 2022). Vision also provides distal access to environmental information, which is often unobtainable via touch (Giudice, 2018; Ottink et al., 2022).

Due to the informativeness of visual information in spatial coding, deprivation of visual information has led to blind individuals coding the space and environment differently than sighted individuals (Battal et al., 2020; Bleau et al., 2023; Gaunet et al., 1997; Giudice, 2018; Millar, 1981; Ottink et al., 2022; Tinti et al., 2006; Ungar, 2018). In terms of the effectiveness of spatial coding, when blind individuals code and process information about smaller-scale environments, such as the space close to the self or a room in a building, they tend to be as effective as sighted individuals (Giudice, 2018; Tinti et al., 2006; Ungar, 2018). However, coding spatial information for a larger-scale space or complex environment, such as an indoor mall or a city, is often more challenging for blind individuals (Giudice, 2018; Ottink et al., 2022; Ungar, 2018).

When coding large spaces, sighted individuals are more likely to code spatial information allocentrically—remembering or recognizing where things are relative to each other—and simultaneously, as they often get a (relatively) huge amount of visual information at the same time. In contrast, blind individuals are more likely to encode information egocentrically—remembering or recognizing locations relative to the self—and sequentially, relying on the temporal order of experiences conveyed through auditory or haptic feedback. (Millar, 1981; Ottink et al., 2022; Pasqualotto & Proulx, 2012; Raz et al., 2007). As a result, blind individuals’ spatial coding of larger spaces often requires greater spatial–temporal integration and more processing resources, which can lead to more difficulties in coding and processing spatial information (Giudice, 2018; Ottink et al., 2022; Tinti et al., 2006; Ungar, 2018). It is interesting to note that blind individuals who adopt spatial coding or processing strategies like sighted individuals often perform within the range of sighted individuals (Ungar, 2018).

Converging evidence shows that both blind and sighted individuals are able to build spatial representations by using unimodal information gathered from different modalities (i.e., visual, auditory, or haptic), as well as multimodal information, even though there are differences in how blind and sighted individuals use different types of information (Battal et al., 2020; Giudice, 2018; Ottink et al., 2022; Tinti et al., 2006; Ungar, 2018). Some researchers suggested that, instead of having separate spatial representations for each modality, we integrate information from different modalities into amodal spatial representations, which are often referred to as “cognitive maps” (e.g., Ottink et al., 2022). Due to the differences in operationalization and measures to assess spatial representations, there is still not a universally agreed-upon definition of “cognitive maps.” Yet, despite the differences in definitions and measures, studies have provided strong evidence for cognitive maps in blind (and sighted) individuals (for further discussion, see Schinazi et al., 2016). It has also been proposed that performance differences in spatial cognition tasks may be best viewed as the result of using different coding strategies, rather than an intrinsic deficit (as was the view in early studies) in spatial cognition in blind individuals (Giudice, 2018; Schinazi et al., 2016; Tinti et al., 2006; Ungar, 2018).

### 3.4. Other Areas in Which Blind Individuals Show Equivalent or Superior Performance to Sighted Individuals

One area that has often been shown to be related to visual–spatial processing in sighted individuals is numerical processing (Crollen et al., 2021; de Hevia et al., 2008; Dormal et al., 2016; Ross & Burr, 2010). Blind children have shown equal or better performance in various numerical tasks, such as reporting the numerical midpoint of a number pair, counting, and mental arithmetic (Crollen et al., 2011, 2021; Crollen & Collignon, 2020). Yet, they are less likely to use their fingers to count and rely less on finger-based number representations, compared to sighted children (Crollen et al., 2011, 2021; Crollen & Collignon, 2020). Blind individuals can achieve high levels of mathematical abilities, and they show brain activations in the classical network of math-related areas during mathematical reasoning (Amalric et al., 2018; Crollen & Collignon, 2020). These findings suggest that visual experience is not mandatory for the development of numerical senses or advanced mathematical reasoning (Amalric et al., 2018; Crollen & Collignon, 2020). And some researchers argue that the number sense is a supramodal, modality-independent faculty (Yang & Collignon, 2025; see also Hofstetter et al., 2021).

It has been suggested that, to compensate for the lack of vision, blind individuals may rely on enhanced auditory working memory for numerical thinking and manipulation (Crollen & Collignon, 2020; Dormal et al., 2016). Furthermore, blind individuals have been shown to have better processing in several aspects of auditory stimuli, such as sound detection and localization, frequency discrimination, relative auditory distance perception, feature memory, and auditory working memory (Battal et al., 2020; Kolarik et al., 2021; Raz et al., 2007; Röder et al., 1999, 2001; Rokem & Ahissar, 2009; Sabourin et al., 2022). They are better at localizing sound in the peripheral or in the horizontal plane (Röder et al., 1999; Sabourin et al., 2022). They are also more efficient in coding auditory information and have better auditory memory (Crollen & Collignon, 2020; Raz et al., 2007; Röder et al., 2001; Rokem & Ahissar, 2009; Sabourin et al., 2022). They use their superior auditory processing and memory to aid in areas where sighted individuals often rely heavily on visual processing, such as spatial and numerical coding (Crollen et al., 2021; Crollen & Collignon, 2020; Dormal et al., 2016; Raz et al., 2007).

Early visual deprivation, even just a few days or months, influences infants’ preferences and perception of faces, which are important social stimuli. The research discussed above illustrates the effects of longer-term visual deprivation and the importance of vision in infants’ fine and gross motor development, social interactions, and how individuals process and represent spatial and numerical information. These findings point to the cascading effects of early visual inputs or lack of visual inputs on later development from infancy continuing through adulthood. Importantly, the studies highlighted in the last section suggest that blind individuals (most studies with adults, but some with children as well) tend to rely on audition to compensate for the lack of vision in areas that are often visually dominated (e.g., numerical representation) in sighted individuals. In the next section, we will turn to the role of audition in early cognitive development.

## 4. Audition

While many may consider vision to be the most dominant sense, audition is key to developing spoken language. Hearing can therefore be considered central to the success of our species, as we communicate primarily through spoken languages around the world. Language and its development are of paramount interest to scientists who study the auditory system. We will not attempt to summarize the vast amount of research on language, as it goes far beyond the scope of this review and has been extensively reviewed elsewhere. Nevertheless, there are other aspects of auditory experiences that have consequences for cognitive development. We will discuss a few examples here, to illustrate the importance of sound as a sensory experience beyond its role in speech. First, we examine the role of music and rhythm in infancy. Next, we will consider the consequences for nonverbal cognitive development in children who are born with no access to sound, namely children with congenital deafness.

### 4.1. Music and Rhythm Are Universal Experiences in Infancy

Many readers may remember the media craze surrounding the “Mozart” effect, which was the popular belief that listening to Mozart could make children smarter. While the “Mozart effect” was unfortunately nothing more than scientific legend (Beauvais, 2015), there is nevertheless a growing body of research which shows that music can positively impact cognitive development, although findings vary in strength, scope, and proposed mechanisms. Starting even before birth, several studies have demonstrated that prenatal exposure to musical stimuli can enhance newborns’ sensitivity to sounds after birth (Arenillas-Alcón et al., 2023; Partanen et al., 2013). These findings suggest that music exposure enhances the perceptual sensitivity to acoustic features of speech, which may have positive consequences for language development (Paula et al., 2023). This is not surprising, as music shares key acoustic features with human speech, and especially with infant-directed speech (Hilton et al., 2022). However, music has also been shown to broadly influence infant cognition beyond its effects on speech. For instance, music entrains infant visual behavior (Lense et al., 2022), influences listening preferences and memory (Saffran et al., 2000), social behaviors (Cirelli & Trehub, 2018; Mehr et al., 2016), and brain activity (Cantiani et al., 2022). Musical training is linked to executive function performance in preschoolers and children (for reviews on this topic, see Table 1).

Partanen et al. (2013) asked whether music can influence brain development even before birth. In the last trimester of pregnancy, mothers in a “learning” group were asked to play “Twinkle Twinkle Little Star” five times per week. At birth and four months later, the infants born to mothers in the learning group (*n* = 17) showed a moderate increase in ERP (event-related potential) amplitude to the familiar melody compared to a control group *(n* = 16). Further, the ERP amplitudes were strongly correlated with the amount of prenatal exposure. These findings suggest that prenatal musical experience may support the formation of long-lasting neural representations, although evidence remains limited to small samples and specific paradigms. Further research could replicate these findings with more robust samples. In addition, the mechanisms underlying the formation of these neural representations, and whether they are unique to music or represent more general effects, are still open questions for future research.

One simple mechanism for the benefits of music on infant cognition could be its regulatory effects. Music has a calming effect on both parents and infants (Cirelli & Trehub, 2020). In a musical adaptation of the Still Face procedure, Cirelli and Trehub (2020) examined the effects of parent singing on distress in 8 and 10-month-old infants (*n* = 68 in each age group). Singing familiar songs helped to soothe distressed infants the most, as well as enhance their attention to singing parents. This effect was small, yet statistically significant. Singing unfamiliar songs was also soothing, though to a lesser extent, while talking alone did not calm infant distress after the Still Face phase (Cirelli & Trehub, 2020). These findings suggest that infant-directed singing may help to regulate infants’ arousal and affect, although observed effects are modest and may not be uniformly stronger than other forms of caregiver engagement. These regulatory effects could indirectly benefit attention and learning, as infants who are calmer and more attentive may then have more opportunities for learning.

Music is also highly rhythmic, and rhythm itself has powerful effects on human behavior and brain activity (Cantiani et al., 2022). A fascinating study by Lense and colleagues investigated rhythmic entrainment as a candidate mechanism for how infant-directed singing can exert powerful effects on infant social cognition and behaviors (Lense et al., 2022). At 2 and 6 months of age, two independent samples of infants (*n* ~ 56 per age group) were presented with audiovisual recordings of nursery rhymes, sung by actual parents. Using eye-tracking, infants’ looking behavior during the songs were recorded and analyzed to test whether gaze fixations became entrained to the rhythmic structure of the songs. As predicted, infants increased their fixations to parents’ eyes in synchrony with the rhythm of their singing (specifically, the strong beats). These findings suggest that social singing in infancy—via rhythmic entrainment—creates optimal learning conditions for social and communicative interactions. Other correlative studies have demonstrated links between rhythm abilities and language, executive function, and motor skills (Frischen et al., 2019; Lau et al., 2022; Tierney & Kraus, 2013). Taken together, this body of research suggests an important link between rhythm and cognition in infants, although much of the evidence to date is correlational and leaves open questions about causality and directionality.

Indeed, another candidate mechanism for how music benefits infant cognition is through social learning. In two experiments by Mehr et al. (2016), 5-month-old infants learned a novel lullaby over two weeks that was either sung to them by a parent, an unfamiliar adult via video call, or via an audio recording inside a toy. In a test session, infants viewed unfamiliar adults singing the learned song or a novel song. Infants selectively attended to the singer of the familiar song, but only when they had learned the song from a singing parent and not from a recording or a video call. These findings suggest that music may acquire a special social meaning for infants under certain learning conditions (Mehr et al., 2016) and mirrors what has been found in the domain of phoneme discrimination (Kuhl et al., 2003), although alternative explanations—such as increased caregiver contingency or familiarity—cannot yet be ruled out. The origin of this specialness is not yet known; one possibility is that the ubiquity of music in infancy means that infants quickly learn to perceive music as an important signal of caregiver attention, through many daily moments of infant-directed singing (Mehr et al., 2019; Yan et al., 2021). One caveat is that, while caregivers around the globe have been found to sing to their infants (Mehr et al., 2019), and their singing shares universal properties (Hilton et al., 2022), the research described here on specific links between caregiver singing and infant cognition is limited to families from Western countries. Therefore, a global perspective on the effects of music on cognitive development is still missing.

A different perspective is that a musical nature could be embedded in our genetic code through evolutionary processes, driven by the dynamics of parent–infant interaction and caregiving demands (Mehr & Krasnow, 2017). Mehr and Krasnow (2017) theorize that if sensitivity to music is implicated in specific genes, then people with abnormalities in those genes will show atypical responses to music. Some provocative evidence for this idea comes from a study by Mehr et al. (2017) that investigated Prader–Willi Syndrome (PWS), a rare genomic imprinting disorder that causes infants with PWS to initially exhibit reduced demands on parental attention (e.g., less crying, less nursing). Indeed, adults with PWS demonstrate atypical responses to music (Mehr et al., 2017), which is in line with Mehr & Krasnow’s ideas. While this evidence from adults with PWS is speculative, the idea that music has become a universal aspect of human biology and cognition is widely discussed, though empirical support for specific evolutionary mechanisms remains indirect (Mehr et al., 2019). Future research could investigate the causal effects of music and rhythm on core cognitive skills in both typical and atypical infant populations across cultures.

Taken together, research on music and rhythm points to multiple plausible pathways through which musical experiences may shape attention, regulation, and learning in development. However, while there is broad agreement that music positively affects cognition, the specific evidence for which aspects of music affect which specific subcomponents of cognition is still scattered (Lu et al., 2025). For example, a recent systematic review by Lu and colleagues examining the effects of rhythm-based training on executive function in preschool-aged children highlighted substantial inconsistencies across studies, a challenge exacerbated by the highly interdisciplinary nature of music–cognition research. They focused on evidence for direct transfer effects on three core executive function skills—working memory, inhibition and cognitive flexibility. Findings revealed only partial support for their hypothesis that rhythm interventions would benefit executive function skills, and highlighted pervasive gaps and inconsistencies in this field. Therefore, despite the apparent consensus regarding positive effects of music on cognition, considerably more research is needed to understand which specific aspects of music convey benefits on cognitive development.

Against this backdrop of emerging findings in typical development, we now turn to what happens when infants are born with no access to auditory information and initially experience a world without sound, as is the case for deaf infants.

### 4.2. Infants with Hearing Loss Show Differences in Visual Cognition

It is not surprising that infants born with profound hearing loss show delays in spoken language development, given that they have little access to rich language input from birth. However, what is surprising is that deaf infants also show differences in their development across a range of cognitive abilities that are not typically associated with hearing. For example, deaf infants seem to be slower at processing static visual images (Monroy et al., 2019) and are slower to learn simple sequences of actions (Monroy et al., 2022). This evidence has sparked debate about the separate roles of sound and language in shaping cognitive and brain development.

The auditory scaffolding hypothesis (Conway et al., 2009) was proposed to explain the unique contribution of sound to domain-general cognition. Under this hypothesis, sound provides temporally ordered information that is vital to the normal development of general sequence processing abilities in infancy. These abilities, in turn, form part of the foundation for key cognitive skills like statistical learning, event perception and memory. In other words, the temporal order in which the acoustic energy from sound is encoded in the human auditory system provides an experience-expectant input for cognitive development. The auditory scaffolding hypothesis is supported by evidence from deaf children or children with hearing loss who demonstrate sequence processing difficulties compared with children who have normal hearing (Conway et al., 2008, Conway et al., 2011; Gremp et al., 2019; Pisoni et al., 2003). However, these specific findings have been contested by studies that have employed tasks that do not rely on verbal rehearsal or have included deaf children of deaf parents who are native signers (Fastelli et al., 2021; Hall et al., 2017b, 2017a; Terhune-Cotter et al., 2021). Therefore, while the auditory scaffolding hypothesis remains actively debated and is not widely accepted as a general account, it is in line with core cognitive theories which consider nonverbal auditory processing skills a central component of intelligence (Flanagan & Dixon, 2014).

Examples of how nonverbal auditory experiences may guide core cognitive skills in early infancy are illustrated in studies on infant motor exploration and action observation. For instance, M. K. Fagan (2019) demonstrated that 9-month-old deaf infants explored novel objects in a fundamentally different way compared to hearing infants (*n* = 16 deaf infants, *n* = 27 hearing infants). While they were just as curious and engaged with the objects, deaf infants sought different sensory experiences with them (for instance, they mouthed rather than banged objects). Presumably, this different pattern of exploration is because they received and were reinforced by other forms of sensory feedback (gustatory, haptic, tactile) rather than auditory feedback. This finding is in line with the many studies that highlight the importance of contingent action-effects for motivating infant actions and their learning about actions (Adam & Elsner, 2020; Elsner, 2007; Elsner & Aschersleben, 2003; Jovanovic et al., 2007; Monroy et al., 2017). Given the many areas in which deaf children’s cognitive development seems to diverge from their hearing peers, these findings also suggest that early sensory experiences may have profound implications for broader aspects of learning and information processing (Bavelier et al., 2006; Codina et al., 2011).

Examining the sensory experiences of infants and children who experience sensory loss—such as congenital deafness—raises fundamental questions about neural plasticity in development. A small number of rigorous neuroimaging and electrophysiological studies have documented cross-modal cortical reorganization in deaf children before and after cochlear implantation (i.e., following partial restoration of auditory input) (Lee et al., 2020; Polonenko et al., 2017; Sharma & Glick, 2016). Together, these findings reveal new information about the rapid timescale and dynamic nature of cortical plasticity associated with early auditory deprivation. Although much remains to be understood about the functional consequences of such reorganization, converging evidence suggests that hearing loss may alter central attentional mechanisms through both bottom-up perceptual and top-down motivational pathways. For example, perceptual input differs in children with hearing loss, resulting in cross-modal changes in the way that cortical neurons are tuned to that perceptual input. The resulting different sensory experiences then shape the interests and goals of infants who are deaf in a different way than babies who are hearing, leading to downstream differences in the development of attentional control. Taken together, this body of work highlights how sensory deprivation can initiate cascading, bidirectional, experience-dependent changes in core cognitive skills like attention.

## 5. Olfaction

Why have taste and smell played a smaller role in developmental sensory research compared with other senses? From a methodological perspective, this neglect has constrained the types of questions that are asked about early cognition, privileging visually and motorically accessible phenomena over sensory systems that are harder to verbalize, isolate, or experimentally manipulate. Both scientists and people in general have historically viewed olfaction as less important to our everyday lives than vision or hearing. For instance, British adults consistently rank smell as the least important of the traditional five senses (Enoch et al., 2019), and in a survey of 7000 teenagers and young adults, about half of them would rather give up their sense of smell than their phone or laptop (McCann Worldgroup, 2011).

However, Majid (2018) considers how the prevailing myth that smell is less relevant to cognition than other senses is reflected in the English language. Smell vocabulary is limited in English and many other languages (Lynott et al., 2020; Majid et al., 2018a), and reference to smell is infrequent compared with other perceptual modalities (e.g., Winter et al., 2018). For instance, children can only name 15% of hazardous smells, but can correctly identify 79% of them, suggesting that our language simply lacks a rich lexicon for smells (Nováková et al., 2018). This could limit a child’s opportunity to learn about smells and smell language. In other words, lack of learning opportunities may explain our difficulties in naming and identifying smells, rather than olfaction being a less relevant sense to human cognition, although other factors like biological salience, task demands, and cultural practices may also contribute.

**Figure 1:**
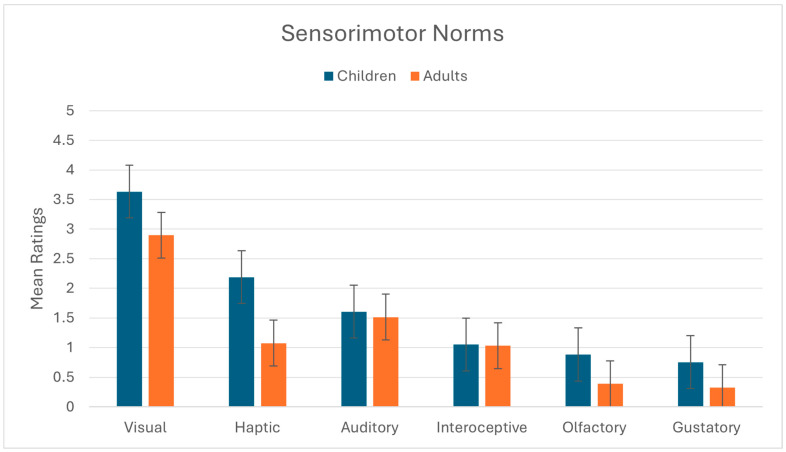
Average ratings of perceptual strength, per sensory modality, for words on the CDI (“children”) and all 40,000 words (“adults”) from the Lancaster sensorimotor strength norms database (Lynott et al., 2020).

A useful way to illustrate this phenomenon is provided by Lynott et al. (2020), who constructed sensorimotor norms for each of almost 40,000 English words, chosen to represent the complete adult vocabulary. For instance, the mean ratings from 0 to 5 for the word “dog” (a word that is typically learned early in infancy) are highest for visual (4.59/5) followed by auditory (4.12/5) and then haptic (3.82/5); olfactory and gustatory trail behind at 2.82/5 and 0.59/5. Their dataset shows that, overall, 74% of words were rated as dominantly visual and less than 1% were dominantly olfactory (dominantly’ in this context refers to the sensory modality with the highest mean rating for that word; in the dog example, dog is dominantly visual). To examine whether this pattern extends to the first words that are typically learned in infancy, we calculated the sensorimotor norms of the subset of words that are included on the MacArthur-Bates Child Development Inventory (MB-CDI; Dale et al., 2023). The MB-CDI is a checklist that generally reflects the first vocabulary words that children learn. Of these 369 words, the mean for olfactory ratings was 0.88, compared to 3.59 for visual ratings. Only one word (flower) in the entire checklist (0.027%) was rated as dominantly olfactory (mean ratings for “flower” are olfactory = 4.39/5, visual = 4.28/5, haptic = 3.33/5, gustatory = 1.17/5, and auditory = 0.28/5). As can be seen in Figure 1, the earliest words that young children learn match the overall adult English vocabulary in terms of the sensorimotor properties that they evoke. This supports Majid’s view that English, in particular, simply does not provide rich learning opportunities about smells. These linguistic limitations have direct consequences for experimental design in developmental research, where tasks often rely on labeling, categorization, or explicit choice, making olfactory cognition difficult to study using standard developmental paradigms.

Further evidence for this linguistic explanation of poor olfactory learning comes from emerging data on the role of smell among the world’s languages. This data suggests that there are languages around the globe that have sizeable smell vocabularies, in contrast to English (Majid et al., 2018b). Crucially, this characteristic is not restricted to languages with few speakers, as previously thought, but has been reported across many hunter–gatherer societies, pastoral and horticultural societies, as well as major languages in industrialized societies (e.g., Cantonese, Thai; Majid et al., 2018a). Smell is even coded in grammar in the Cha’palaa language, spoken in Ecuador (Floyd et al., 2018). The more prominent role of olfactory language in those societies implies that their children are more often exposed to complex olfactory language from early on. These examples are consistent with other studies that have revealed the fascinating ways in which language can affect how we code and represent perceptual input (Winawer et al., 2007). Future research is needed to determine whether this exposure results in stronger associations between smell and other modalities, or better learning for representations that contain or overlap with smells.

### 5.1. Smell Is Important for Memory from Early in Infancy

A small number of studies on the overlap between olfaction and key cognitive domains are broadly consistent with such a hypothesis. There is a sizeable body of literature on this overlap that focuses on memory, and the role of olfactory context in supporting memory (Delaunay-El Allam et al., 2010; Jehl & Murphy, 1998; Schroers et al., 2007; Suss et al., 2012). For instance, Schroers et al. (2007) asked whether olfactory context can trigger memory retrieval in three-month-old infants, as visual and auditory contexts have been shown to do. Infants were exposed to a specific odor (cherry, coconut, or no odor) while being trained in a version of the classic mobile conjugate reinforcement task (Rovee & Fagen, 1976). At test, infants who were exposed to the same odor either 1 or 5 days after training demonstrated memory retention for the mobile paradigm, whereas infants exposed to a different, or no odor at test showed no memory retention.

The authors suggest that the specific finding of 5-day post-training memory retention reveals that odor context is fundamentally different from visual or auditory cues, which do not elicit retention at longer intervals (Fagen et al., 1997; Rovee-Collier & Sullivan, 1980). Instead of serving just as a contextual cue, the odor may become an integral part of the infant’s conceptual representation of the mobile itself (in the authors’ words, it becomes a “smelly mobile”; p. 686). However, alternative explanations for these findings are that the odor indeed simply supports memory via encoding specificity by serving as a contextual cue, or by increasing infants’ general arousal or attentional state. Both possibilities could explain enhanced retention independently of the odor becoming part of the mobile representation. Nevertheless, there are also anatomical and neurophysiological explanations for why odor may become a more integral part of an infant’s newly learned representation than visual or auditory cues, such as direct connections between the olfactory cortex and memory circuitry in the brain (Chu & Downes, 2002). In summary, odor may play a central role in the development of infant memory, although more research is needed to confirm whether this role is unique among the senses.

### 5.2. Infants’ Earliest Social Preferences Are Modulated by Smells

The idea that olfaction is uniquely important to the earliest aspects of infant cognition is supported by a separate strand of research on the role of olfaction on prenatal and neonatal development. Much of this work has focused on breastfeeding because of its relevance for neonatal survival but has nevertheless yielded important insights into the origins of infant social cognition. Olfactory perception likely begins functioning in the third trimester of pregnancy, during which infants are bathed in amniotic fluid that is full of odorous compounds (Schaal, 2016). Research into the odor preferences of newborns has revealed that infants are highly sensitive to breastmilk odors and can learn to discriminate among them quickly and flexibly (Balogh & Porter, 1986). For instance, 2-week-old infants who are breastfed prefer their mother’s odor over that of another woman, whereas 2-week-old infants who are bottle-fed do not. Further, breastfed infants prefer the smell of their own mother’s milk over that of another woman’s breastmilk, whereas bottle-fed infants do not prefer the smell of their normal brand of formula over that of another brand (Macfarlane, 1975).

What does this have to do with early social cognition? Researchers have investigated the link between early odor learning and its effects on social learning in infancy. One important marker of infant social cognition is attention to faces—infants have been shown to prefer face-like stimuli even *before birth* (Reid et al., 2017). Early facial recognition abilities in young infants are robust and are reflected in specialized brain activity for faces (de Haan et al., 2002; J. F. Fagan, 1972), leading to the widely held view that human faces are treated as special by our cognitive system from the earliest moments in life. A fascinating study by Durand et al. (2013) asked whether exposure to their mother’s odor can selectively influence an infant’s attention to faces. Inspired by research on odor–vision interactions in adults, these researchers investigated whether olfaction modulates infant attention to faces. Four-month-old infants (*n* = 48) were presented with pictures of faces or cars that were paired with a social smell: the body odor of their mother. Results showed a clear odor–stimuli interaction: infants looked more to the faces than the cars when paired with their mother’s smell (worn t-shirts), but not when paired with a control smell (unworn t-shirts). Even their distribution of gaze to the faces was affected by smell, as infants looked more specifically to the eye region compared to mouth, nose or other facial areas, in the smell condition.

These findings reveal that social odors can strengthen infants’ preferences for faces and eyes under certain experimental conditions, even when those faces are unfamiliar. Like the “smelly mobile” interpretation, these authors suggest that infants are integrating olfactory and visual information. This integration is likely based on prior experience with similar odor–vision links in their daily life (i.e., they typically see and smell other people in their environment). Odors are therefore a socially relevant signal that modulates infant attention. More recent studies expanded this work using neuroimaging techniques to confirm the finding that odor influences face processing and categorization (Düfeld et al., 2025; Kiseleva et al., 2024; Leleu et al., 2020). However, while the effects in Durand et al. (2013) were large, the study did not include a control condition featuring another biological odor source such as an unfamiliar female smell. Therefore, research to further extend and replicate these findings would strengthen the evidence for links between olfaction and face perception.

### 5.3. Smells Guide Motor Exploration

Olfaction has even been found to potentially influence infant object exploration. As discussed earlier, motor exploration is a key milestone that reflects the combined influences of cognitive, motor, and perceptual development. Attending to and exploring object properties offers crucial sensorimotor experiences that help infants begin to learn about object affordances, causal relationships, and perception–action contingencies (M. K. Fagan, 2019). A study by Durand et al. (2008) demonstrated that adding a novel, non-food-related scent (violet) to a toy changes infants’ exploratory behaviors with those toys. Interestingly, the direction of this change was unexpected: infants played with and attended to the scented toy *less* than an unscented toy. These findings suggest that infants’ smell preferences can significantly influence their manual and visual exploratory behaviors, although the direction and robustness of this effect remain unclear. However, we note that these findings come from a single study with a small sample (*n* = 16) representing a relatively wide age range (7–15-month-olds). Given the importance of motor exploration for the development of core cognitive skills like joint attention (Yu & Smith, 2017), social interaction (Monroy et al., 2024), and even vocabulary development (Yu et al., 2019), further research on the influence of smell on motor behavior is warranted.

## 6. Taste

If there is relatively little research on the sensory domain of olfaction in the cognitive development literature, there is even less on gustation. As eloquently stated in Wiggins and Keevallik (2021), “taste has for the most part been relegated to the unanalyzable interiority of the individual and ignored in much of the academic literature on the senses” (p. 1). Along with olfaction, it appears that academics and the public have generally associated gustation with the more primal aspects of human nature, rather than a sensory modality deserving of the serious scientific inquiry that we devote to vision or audition. In addition, the relative neglect of olfaction and gustation may reflect measurement limitations, as these senses are more difficult to standardize, quantify, and isolate experimentally than vision or motor behavior within conventional developmental paradigms.

Most research on taste in infants is devoted to understanding infant feeding behaviors and the development of taste, as in olfactory research. In toddlers and young children, there have been many studies devoted to understanding their notoriously “picky” feeding behaviors, and how they relate to food preferences, self-regulation and executive function, and long-term health (Keim et al., 2021; Nicklaus & Schwartz, 2019; Paroche et al., 2017; Russell & Russell, 2020). However, the research on the development of taste preferences indirectly suggests that taste is as connected with developing cognitive domains as other sensory modalities. For instance, Paroche et al. (2017) conducted a systemic review of research on how infants and children learn about food. Their review revealed four major developmental learning processes associated with learning about food: familiarization, observational learning, associative learning, and categorization. These processes have been well described in the literature as core cognitive skills in infancy (Rakison & Oakes, 2003). While Paroche et al. (2017) was focused on understanding how these skills contribute to early eating behavior, there are likely bidirectional links such that early taste experiences contribute to the development of those core cognitive abilities, although direct evidence for this remains limited. For instance, repeated multimodal experiences of tasting, handling, and looking at foods and objects associated with feeding may enhance infants’ early conceptual understanding and core learning mechanisms (Meng et al., 2023).

One fascinating study did put taste at the center of their investigation on infant sensory experiences. Wiggins and Keevallik (2021) conducted a qualitative study on the multimodal, coordinated, and embodied experience of tasting during infant mealtimes. Tasting is approached as a central sensory experience embedded within parent–infant interactions and embodied social interactions. Through microanalyses of infant’s feeding interactions—for instance, being spoon-fed new foods by their caregiver—they document how infant tasting experiences are accompanied by actions, facial expressions, mutual gaze, and vocalizations, most notably “mmm”. These interactions all provide highly salient, multimodal sensory experiences and opportunities for learning. Caregiver manual actions and vocalizations serve to initiate and prolong infant tasting and their focus on tasting. This analysis suggests that the experience of tasting may play a more central role than previously considered in infant everyday actions and interactions. It also mirrors findings from similar analyses on parent–child play, in which caregiver multimodal behaviors have been shown to support infant sustained visual attention and language (Yu & Smith, 2012, 2016, 2017). However, this speculation currently remains an open empirical question.

There are a few other examples of studies that investigate the importance of mealtime contexts in the development of infant cognitive skills. For instance, Nonaka and Goldfield (2018) investigate the emergence of tool-use skills during mealtimes. Other studies have investigated mouthing as a type of exploratory behavior during play (M. K. Fagan, 2019; Orr, 2021), yet these studies typically do not treat taste as a central analytic dimension of those behaviors. We note that this limitation also applies to our own ongoing work, which examines the dynamics of parent–child interactions during mealtimes to explore the effects on infant attention (Kaplan et al., 2026). However, the studies described here do not consider taste (or smell) as a part of these experiences. Notably, taste is rarely treated as a cognitive variable in its own right. Instead, it is relegated to the background of feeding behavior, leaving open critical questions about how gustatory experience contributes to attention, learning, and representation in infancy. Instead, the focus is exclusively on infants’ and caregivers’ visual, auditory, and motor actions and performance.

## 7. Discussion

In this review, converging evidence across sensory modalities suggests that learning is supported by infants’ sensitivity to structured regularities in sensory input, whether these regularities are temporal (audition and motor), spatial (vision and motor), or socially contingent (vision or olfaction in caregiver–infant interactions). At the same time, the reviewed literature reveals important divergences across modalities, particularly in developmental timing and functional role: for instance, auditory and motor systems appear to scaffold early prediction and sequencing, whereas olfaction and gustation may play a larger role in infants’ earliest social, emotional, and memory-related representations. These findings suggest that our senses each make unique contributions to infant cognition. At the same time, the reviewed evidence points to several potential overlapping mechanisms in how sensory–motor experiences drive cognitive development.

Throughout this review, we have used cognitive terms such as *attention*, *memory*, *prediction*, and *social cognition* in a deliberately non-unitary sense. Both empirical findings and theoretical frameworks indicate that these constructs are made up of multiple underlying processes that differ in their representational format, temporal structure, and task demands, and that are often shaped by the properties of specific sensory and motor experiences. For example, attention may refer to sustained visual engagement, auditory orienting, or socially coordinated joint attention; memory may involve spatial, event-based, or person-linked representations; and social cognition may rely on visual, auditory, or olfactory cues depending on context. Different sensory experiences likely scaffold different subcomponents of broader cognitive domains. Even when multiple senses support the same cognitive constructs (e.g., spatial representation or prediction), they may do so via different processes. For instance, vision may support memory through spatial integration, whereas audition supports memory through temporal integration and olfaction supports memory through affective mechanisms and associative learning.

### 7.1. Predictive Processing and Sensory Experiences

One common thread that can be identified across the evidence collected on each sensory modality is the role of prediction. Predictive processing is a prominent theoretical framework in psychology and cognitive sciences (Köster et al., 2020). Predictive processing is considered a basic working principle of how the human brain works (Friston, 2005, 2010). This principle is that the brain is continuously faced with incomplete, ambiguous and noisy data from the signals received through the senses about the environment. To deal with the uncertainty generated from these signals, the brain generates predictions about the most likely incoming input and uses prediction errors to refine those predictions and optimize internal representations of the environment.

It is increasingly recognized that predictive processing’s explanatory power extends to key aspects of infant learning (Köster et al., 2020; Nagai, 2019). Here, we draw on predictive processing as an interpretive framework to synthesize patterns that emerge across the reviewed literature. Much of the research we have described in this article is compatible with this framework, whether explicitly or implicitly. For example, we have described research supporting the idea that music entrains infant visual attention and social cognition. Prediction may be one mechanism that contributes to this process, as caregivers make themselves highly predictable when singing to their infants and regular beats and rhythms are highly predictable. Prediction could be a general mechanism through which rhythm and music exert powerful effects on cognitive development (Frischen et al., 2022).

One crucial open question regarding predictive processing framework is, if the brain operates by generating predictions based on an internal model of the world, what is the origin of those predictions? How does the infant brain generate its first prediction, and what information does it use? Given the research we have outlined on the important role of olfaction (and, probably, gustation) on infants’ earliest representations and behaviors, a compelling possibility is that taste and smell might contribute to the formation of infants’ earliest predictive models.

### 7.2. Sensory Processes in a Dynamic System

The dynamic systems account considers how general principles of biological self-organization can be applied to understand the developmental process in infants. From a systems view, observed behaviors are the product of the organism’s patterns of responses to and interactions with multiple forces in their environment (Thelen & Smith, 2007). A similar theory is the developmental cascades framework, which focuses on how interactions across multiple domains and timescales shapes development, invoking Waddington’s famous metaphor of a ball rolling downhill and being “nudged and swayed by divots and valleys until it reaches a destination” (Schneider & West, 2025). In his book, *The Dynamics of Behavior Development: An Epigenetic View*, Kuo (1976) called for scientists to study a broader range of events in the environment and of the organism to understand behavior: “[W]e must take quantitative measures of stimulative effects of every sensory modality, and make qualitative analyses of the interactions of the component parts of the environmental context or complex” (p. 190).” In other words, Kuo advocated for a broader examination of sensory and environmental factors that may not be obvious contributors to emerging behaviors. This systems view was overshadowed by the mainstream theories of that time, such as Bowlby (1969) and Piaget (1952).

However, a systems approach to developmental science has nevertheless yielded crucial insights into our understanding of infant development. For instance, work by Goldstein and West showed that infant babbling is shaped through the interdependencies between mother’s reactions to babbling and the feedback from their own vocalizations, rather than driven solely by articulator maturation (Goldstein et al., 2003). More recent work has revealed the complex ways in which caregiver behaviors and infant sensory capacities interact to shape visual attention (Yu & Smith, 2016), word learning (Yu & Smith, 2012), and motor development (Adolph & Hoch, 2019; Iverson, 2022). However, Kuo’s call from 1967 has yet to be fully addressed: olfaction and gustation are not as well represented in the research on infant sensory systems and how they contribute to the overall dynamics of the system and its environment. As we saw earlier, odors drive fundamental infant behaviors and regulatory processes from even before birth (Schaal & Durand, 2012). Odors steer arousal states, control infant distress responses, elicit voluntary and involuntary actions, and shape infants’ earliest learned associations. Olfaction and gustation could trigger the earliest reactions, processes and behaviors in the infant, paving the way for more complex abilities and behaviors to emerge. Just as developmental researchers now acknowledge that infant development cannot be understood outside of cultural context, perhaps future research will reveal that odor context and taste experiences are just as integral.

One relevant and critical open question concerns the developmental timing of sensory influences on cognition. While sensitive periods have been well characterized for audition (Houston & Miyamoto, 2010) and vision (Lewis & Maurer, 2005), there is less comparable evidence for olfaction and gustation (though see Mennella & Castor, 2012 and Romantshik et al., 2007). This asymmetry limits our ability to compare developmental plasticity across modalities and highlights the need for further studies of underrepresented sensory systems.

### 7.3. Taking Culture into Account

Children with different sensory–motor capacities have different learning opportunities, either because their sensory–motor abilities allow them to sample information and interact with the world differently or because their abilities affect how social partners interact with them (Campbell & Johnston, 2009; M. K. Fagan, 2019; Karasik et al., 2014; Soska & Adolph, 2014). Importantly, children live in different cultural environments; and different cultures provide different sensory–motor learning opportunities and shape the experiences children have (Karasik et al., 2023; Majid et al., 2018a). For example, infants raised in Tajikistan with gahvora cradling experiences have limited motor practice opportunities compared to infants raised in many other cultures (Karasik et al., 2023). On the other hand, children learning Thai or Cantonese have richer input in smell-related language compared to English-learning children and, thus, may have more opportunities to learn to talk about or distinguish between smells (Majid et al., 2018a). Cross-cultural differences, as well as within cultural variations, in feeding and eating behaviors affect children’s preferences and learning of food tastes, smells, and textures (e.g., more exposure usually leads to higher preference; Nicklaus & Schwartz, 2019; Paroche et al., 2017). Similarly, cultural differences in musical structures affect young children’s musical preference and perception (Arrasmith, 2020; Soley & Hannon, 2010). Cultural differences in visual environments have been shown to affect adults’ grouping and preferences for landscapes (Petrova et al., 2015). It is worth investigating whether, and if so, how cross-cultural differences and within-cultural variations (e.g., socioeconomic or reginal variations) in visual and auditory environments affect children’s preferences and categorization of different visual and auditory stimuli. An increasing number of studies look at parent and child media and technology use on different aspects of child development (Linder et al., 2021; Mannell et al., 2024). Different cultures, societies, or families with different socioeconomic or educational backgrounds likely provide different opportunities for media and technology use. One further question to ask then is whether different cultures affect cognitive development by providing infants with different sensory–motor experiences.

Most studies on how sensory–motor experiences affect cognitive development were conducted in the WEIRD cultures (Adolph & Hoch, 2019; Adolph & Robinson, 2015; Nielsen et al., 2017). What we see as the “norms” or “standards” in children’s development tend to be what are typical in the WEIRD cultures and are often biased by researchers’ backgrounds and where the samples are drawn from (Adolph & Franchak, 2017). These norms or standards are not just used in research but often in clinical or educational settings as well. For instance, parents in WEIRD cultures are routinely told to talk and read regularly to their children, because early vocabulary skills are considered a critical milestone in these societies. On the other hand, activities or behaviors that are rare or not generally practiced in the WEIRD cultures (e.g., a 5-month-old infant sitting independently on adult furniture without an adult nearby or a young child carrying heavy loads, navigating different terrains, or riding a horse) often do not get studied and are never included in developmental assessments. Yet, these different practices or experiences may lead to different developmental trajectories and affect the ages when children achieve certain developmental “milestones.” Taking different cultures and practices into account will allow us to have a more comprehensive understanding of children’s development.

Even though we see a rise in the number of developmental studies conducted in non-WEIRD cultures over the past decade or so, the role of culture in shaping children’s learning opportunities, experiences, and outcomes is still a relatively unexplored topic. Whether differences in early cultural experiences have cascading effects either in the short term or long term, and whether different developmental pathways lead to the same or different outcomes, are exciting topics for further exploration (Oakes, 2023).

### 7.4. Taking a Difference Perspective, Instead of a Deficit Perspective

Another lesson we have learned from the studies reviewed here is that instead of viewing the lack of certain type of sensory–motor experience (e.g., lack of vision or hearing) as a deficit, an alternative is to take a Difference perspective and ask how lacking certain type of sensory–motor experience may affect how we sample and process information and how it affects the strategies we use when interacting with the world. For example, understanding the fundamental differences between how we gain spatial information from different senses will allow us to understand why acquiring spatial information tends to be more resource-demanding in blind individuals compared to sighted individuals (Giudice, 2018; Ottink et al., 2022; Tinti et al., 2006; Ungar, 2018). Vision allows for simultaneous intake of a large amount of information with relatively high precision of object locations compared to touch, which allows for sequential intake of information, or hearing, which only provides the general direction of object/sound locations. Using touch or hearing to gain spatial information therefore poses a higher demand on memory and spatial–temporal integration and can then lead to differences in spatial learning and performance in blind and sighted individuals. Changing from the Deficit perspective to a Difference perspective can help us rethink what type of information sampling/processing strategies or experiences are required for more efficient and effective performance and may lead to better designs of assistive devices and training programs for individuals lacking certain sensory–motor experiences (Giudice, 2018). To some degree, this proposal resonates with the interactionist/ecological neurodiversity framework, which argues that “disability is the product of an interaction between the characteristics of a disabled person and the environment around them” (Dwyer, 2022, p. 77, see also Pellicano & den Houting, 2022; Sonuga-Barke & Thapar, 2021). According to this framework, disability can be addressed by reshaping the environment (e.g., by making things more accessible or shaping the environment to fit an individual’s needs) or by changing an individual (e.g., by equipping an individual with adaptive skills or assistive devices).

This Difference perspective is also in line with the proposals of the dynamic systems approach and developmental cascades that we should take variabilities and interactions into account when looking at development (Oakes, 2023; Smith & Thelen, 2003). Different sensory–motor capacities affect the information children receive and the types of strategies they use to process the information. Some strategies may be more efficient or effective than others. Variations in children’s capacities and experiences at different timescales do not only influence how children interact with the world but also how the world interacts with them (Chen et al., 2020; Kretch et al., 2014, 2025). Moment-by-moment changes in children’s bodily experiences create different signals and learning opportunities (Yu & Smith, 2012, 2017). It is therefore important to take the dynamics of children’s embodied experiences and the resulting variations in children’s interaction with the physical and social environments into account (Hirai et al., 2020; Musculus et al., 2021). These different factors can further interact with each other and lead to different developmental pathways.

### 7.5. Moving Forward

In this review, we have discussed how early sensory–motor experiences affect cognitive development. We focused on one modality at a time and elaborated on how each type of experience can shape early cognitive development. However, one thing we did not include in the review is how multisensory or intersensory interactions may affect early development. Sensory input from one modality can affect our perception or processing in another modality. For example, vision has been shown to provide a frame of reference for multisensory spatial representation that involves the use of auditory, tactile or proprioceptive perceptions (Cappagli et al., 2017; Röder et al., 2004). And our sense of taste has long been thought to be strongly influenced by the sense of smell (Spence, 2015a; but also see Stinton et al., 2010). Recently, it has been shown that vision, audition, and touch can also affect our flavor perception (Spence, 2015b). How these multisensory or intersensory interactions affect cognitive development is an open and exciting area for future research.

Although the present review does not aim to provide clinical guidelines or caregiver recommendations, the findings reviewed here have important implications for research on the development of treatment or intervention programs. For example, motor learning, such as learning dance sequences or rock climbing, often involves skills that include prediction, planning, and sequence learning. Typically developing young children and certain clinical populations, such as children with dyslexia or developmental language delays (DLD) have been reported to struggle with these skills (Hsu & Bishop, 2014; Orban et al., 2008). An important question for future research is whether motor learning can be used in training these skills in both typically developing and clinical populations. Another example is whether the calming or attention-getting effects of rhythmic patterns in child-directed music (e.g., nursery rhymes) are restricted to the auditory domain or whether they can be found in other modalities, such as the visual or tactile domains (Lense et al., 2022). An important but unexplored research question is whether rhythmic signing produces similar regulatory effects for deaf infants. Finally, early sensory–motor behaviors may also offer promising avenues for identifying early markers of atypical cognitive development. For instance, a lack of preference for affiliative interactions in infancy has been suggested as an early marker for atypical socio-moral development (Geraci et al., 2025). Future research could examine whether specific patterns of sensory–motor behaviors predict later cognitive outcomes, and whether early interventions targeting these behaviors can mitigate the resulting difficulties. Answering questions like these may help to bridge basic sensory research with important practical implications.

Child development is multifaceted and involves rapid changes in multiple domains; these changes are often multi-causal (Oakes, 2023; Smith & Thelen, 2003). As we mentioned in the introduction, the increasing specialization within developmental science results in siloes of expertise and can sometimes lead to narrowly focused views and interpretations. In this review, we brought together different areas of research and highlighted some common themes on how different sensory–motor experiences may affect cognitive development. We believe taking a whole-child view does not only allow us to have a comprehensive understanding of development. It can also open exciting new avenues for developmental research.

## Figures and Tables

**Table 1:** Selected systematic review or critical review papers on the relationships between sensory–motor experiences and cognitive development. To our knowledge, there are no such review papers on olfaction.

Sensory–Motor Domain	Authors (Year)	Topic
Motor	Shimko and James (2025)	The Relationship Between Motor Development and ADHD
Motor	Athanasiadou et al. (2020)	Early motor signs of attention-deficit hyperactivity disorder
Motor	Jongbloed-Pereboom et al. (2012)	Motor learning and working memory in children born preterm
Motor	van der Fels et al. (2015)	The relationship between motor skills and cognitive skills in 4–16-year-old typically developing children
Motor	Zeng et al. (2017)	Effects of physical activity on motor skills and cognitive development in early childhood
Vision	Grumi et al. (2021)	The interaction between visually impaired children and their parents
Vision	Pasqualotto and Proulx (2012)	The role of visual experience for the neural basis of spatial cognition
Vision	Sepúlveda-Palomo et al. (2025)	Verbal and spatial working memory capacity in blind adults and the possible influence of age on blindness onset
Vision	Schinazi et al. (2016)	Spatial navigation by congenitally blind individuals
Audition	McFayden et al. (2023)	Verbal and visual serial-order memory in deaf signers and hearing nonsigners
Audition	Lima et al. (2023)	Neurocognitive function in children with cochlear implants and hearing aids
Audition	Lu et al. (2025)	Music training and executive function in preschoolers
Audition	Rodriguez-Gomez and Talero-Gutierrez (2022)	Music training and executive function in children
Gustation	Paroche et al. (2017)	How infants and young children learn about food

## Data Availability

The data presented in this study were derived from the following resources available in the public domain: the Lancaster Sensorimotor Norms database (https://www.lancaster.ac.uk/psychology/lsnorms/, accessed on 1 July 2025) and Wordbank (https://wordbank.stanford.edu/, accessed on 1 July 2025).
